# Internal gradient distributions: A susceptibility-derived tensor delivering morphologies by magnetic resonance

**DOI:** 10.1038/s41598-017-03277-9

**Published:** 2017-06-12

**Authors:** Gonzalo A. Álvarez, Noam Shemesh, Lucio Frydman

**Affiliations:** 1 Department of Chemical Physics, Weizmann Institute of Science, Rehovot, 76100 Israel; 2 Centro Atómico Bariloche, CONICET, CNEA, 8400 S. C. de Bariloche, Argentina; 3 Champalimaud Neuroscience Programme, Champalimaud Centre for the Unknown, Lisbon, 1400–138 Portugal

## Abstract

Nuclear magnetic resonance is a powerful tool for probing the structures of chemical and biological systems. Combined with field gradients it leads to NMR imaging (MRI), a widespread tool in non-invasive examinations. Sensitivity usually limits MRI’s spatial resolution to tens of micrometers, but other sources of information like those delivered by constrained diffusion processes, enable one extract morphological information down to micron and sub-micron scales. We report here on a new method that also exploits diffusion – isotropic or anisotropic– to sense morphological parameters in the nm-mm range, based on distributions of susceptibility-induced magnetic field gradients. A theoretical framework is developed to define this source of information, leading to the proposition of internal gradient-distribution tensors. Gradient-based spin-echo sequences are designed to measure these new observables. These methods can be used to map orientations even when dealing with unconstrained diffusion, as is here demonstrated with studies of structured systems, including tissues.

## Introduction

Nuclear magnetic resonance (NMR) exploits the magnetic properties of nuclei to monitor the environments of molecules and tissues. Thanks to its non-invasive nature, NMR is widely used in medical, biological, chemical and condensed matter research^1^. In its *in vivo* setting this is mainly done using magnetic field gradients, leading to magnetic resonance imaging (MRI) methods widely used to image tissues by monitoring the signal emitted by the protons of water. Signal sensitivity and diffusion considerations usually limit MRI’ s spatial resolution to tens of micrometers; however, the fact that the observed nuclear spins can report on their environment by sensing other interactions, is often exploited to attain a higher resolution^1–5^. In particular, in structured systems including microporous materials and living tissues, the displacement restrictions undergone by water molecules driven by Brownian or forced motions, are commonly used to monitor the sizes and shapes of the cavities where water moves^6–17^. Several techniques that exploit these diffusion-driven effects are available, enabling to attain *μ*m and sub-*μ*m resolution^14, 18–21^. These techniques have to deal with the challenge of how to obtain sub-voxel information from the voxel-averaged values that MRI senses. This usually requires modeling the ensemble of microstructures with suitable size and displacement parameters that define probabilistic distributions^22, 23^; with this modeling an enhanced resolution and valuable contrasts reporting about size and shape distributions can be extracted, which considerably enhance the value of NMR and MRI exams.

Another effect that can enhance the morphological information about objects placed in a magnetic field, originates from mapping electromagnetic properties at a very local level. For anisotropic objects placed in a static magnetic field, susceptibility-induced effects can serve as such source of microscopic structural information^24–33^. *μ*m-sized morphologies have thus been extracted based on mapping magnetic susceptibility tensors^34^ and relaxation times ${T}_{2}^{\ast }$ ^35, 36^; usually, however, these susceptibility- and dipolar-based mappings require the rotation of the sample being studied with respect to the main magnetic field, in order to deliver their full complement of microscopic morphological information^28, 34, 37^. Still, susceptibility tensor imaging techniques are attracting increasing attention, particularly within the ultrahigh field *in vivo* MRI realm^28, 38–42^. A recent study suggested that these effects could be used to probe orientations in heterogeneous media without resorting to field rotation plots, by measuring the *width* of the internal gradient-distributions originating from these susceptibility effects^43^. This width was suggested to constitute a symmetric tensor, whose smallest principal component would have an eigenvector oriented along – and therefore would mark— the main axis of an anisotropic structure. The present study formalizes these effects within the framework of a new internal gradient-distribution tensor (IGDT) formalism, leading to a susceptibility-derived observable whose determination with the aid of free or constrained diffusion, can pinpoint the distributions of oriented domains in the nm-mm range. IGDTs are related to the second moments – i.e., the local distributions– of the internal field gradients felt by the spins, and it is possible to measure them by applying external gradients at several orientations, without having to physically reorient the sample. Measuring the IGDTs, however, is not straightforward, as their effects may be masked by other sources of spin decoherence – including those arising from diffusivity, *T*_2_, ${T}_{2}^{\ast }$ and pulsing/gradient/timing experimental imperfections. A novel NMR sequence is thus developed to detect the IGDTs, based on the selective dynamical recoupling concepts of diffusion effects^20, 21, 44^. Using this method it is shown that structure orientations in tissues and objects can be mapped, in proof-of-principle demonstrations involving *ex-vivo* pig spinal cords and a “U-tube” phantom.

## Results

### Susceptibility-induced internal gradient-distribution tensors

To visualize how IGDTs arise and how they can be measured, consider the normalized magnetization arising from an ensemble of non-interacting and equivalent, freely evolving spins in a liquid: $M(t)=\langle {e}^{-i\varphi (t)}\rangle$, where the brackets account for an ensemble average over the evolution phases *ϕ*(*t*)^4, 5, 45–47^. For sufficiently long transverse relaxation times *T*_2_ the decay of this evolving magnetization will be dominated by bulk magnetic field inhomogeneities; however, by applying spin-echo sequences, one can refocus these global dephasings, and probe the random molecular motions that at a microscopic level spins perform within these inhomogeneities. Indeed, while a spin-echo sequence makes the average phase $\langle \varphi (t)\rangle$ arising from the ensemble zero, a random-walk diffusion will impart a non-zero variance to the phase *ϕ*(*t*) given by a Gaussian statistics^48^; i.e., will make $M(t)=\exp \{-\frac{1}{2}\langle {\varphi }^{2}(t)\rangle \}$. Consider then the decay of this magnetization due to diffusion processes occurring while under the action of external magnetic field gradient $\vec{G}$, acting in unison with an internal microscopic field gradient ${\overrightarrow{G}}_{0}$. At a given spin-echo time *TE*, the spin signal decay can be characterized by an attenuation factor $\beta (TE)=\frac{1}{2}\langle {\varphi }^{2}(TE)\rangle$. As described elsewhere^4, 5, 45–47^ this *β*(*TE*) will be given by the overlap between the diffusion spectrum tensor **D**(*ω*), and a filter function ${\vec{{\mathscr{G}}}}_{{\rm{tot}}}(\omega ,TE)$ given by the Fourier transform of the time-dependent total-gradient ${\overrightarrow{G}}_{{\rm{tot}}}(t)=\overrightarrow{G}(t)+{\overrightarrow{G}}_{0}(t)$. The formalism leading to this conclusion assumes that the stochastic diffusion tensor is expressed in terms of its spectral components, and that both the applied and the internal gradients *G*, *G*_0_ are time-dependent vectors. The modulation of the external gradient $\overrightarrow{G}(t)$, can be summarized by an overall strength $\overrightarrow{G}$ and by a time dependent modulation waveform *f*(*t*), −1 ≤ *f*(*t*) ≤ 1. ${\overrightarrow{G}}_{0}$, the susceptibility-induced background gradient being sought, is in principle constant; it can, however, be modulated by radio-frequency (RF) pulses on the spins, leading to a characteristic modulation function −1 ≤ *f*_0_(*t*) ≤ 1. The overall gradient is thus ${\overrightarrow{G}}_{{\rm{tot}}}(t)=\overrightarrow{G}\,f(t)+{\overrightarrow{G}}_{0}\,{f}_{{\rm{0}}}(t)$, and the attenuation factor takes the form^4, 5, 45–47^.

1 $$\beta (TE)=\tfrac{{\gamma }^{2}}{2}{\int }_{-\infty }^{\infty }d\omega \,{\overrightarrow{{\mathscr{G}}}}_{{\rm{tot}}}^{\dagger }(\omega ,TE)\cdot \frac{{\bf{D}}(\omega )}{{\omega }^{2}}\cdot {\overrightarrow{{\mathscr{G}}}}_{{\rm{tot}}}(\omega ,TE\mathrm{).}$$

Here ${\overrightarrow{{\mathscr{G}}}}_{{\rm{tot}}}(\omega ,TE)=\overrightarrow{G}\,F(\omega ,TE)+{\overrightarrow{G}}_{0}\,{F}_{0}(\omega ,TE)$, with the filter functions *F(ω*, *TE*) and *F*_0_ *(ω*, *TE*) given by the Fourier transforms o*f f*(*t*) and *f*_0_(*t*) respectively^20, 21^.

It follows from Eq. (1)’s bilinear form that the magnetization’s attenuation factor comprises three terms^49, 50^ (cf. Supplementary Information 1)

2 $$\beta (TE)={\beta }_{{G}^{2}}(TE)+{\beta }_{{G}_{0}^{2}}(TE)+{\beta }_{\overrightarrow{G}\cdot {\overrightarrow{G}}_{0}}(TE\mathrm{).}$$

**Figure 1:**
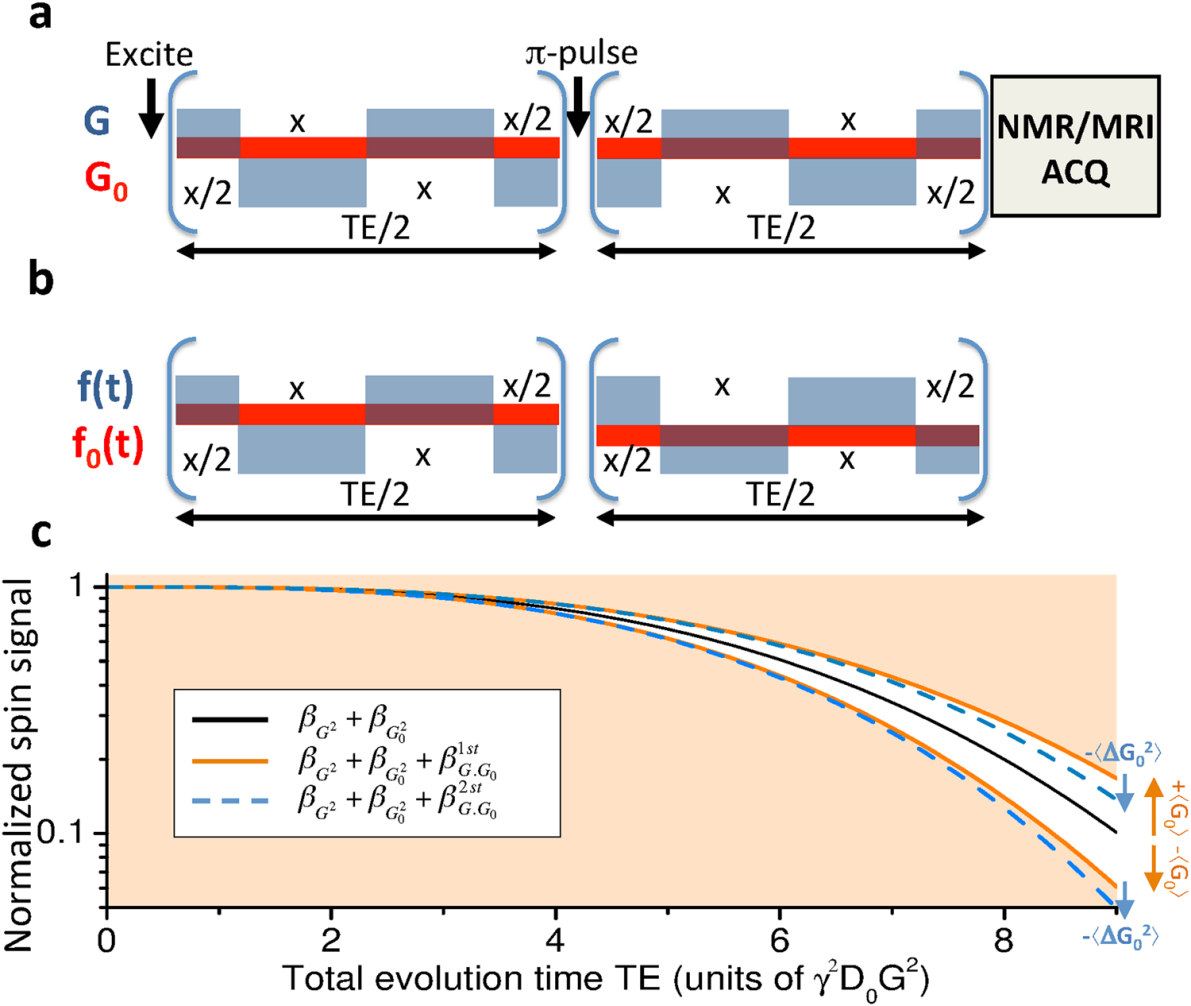
Defining and measuring the effects of susceptibility-induced gradients-distribution tensors by magnetic resonance. (**a**,**b**) Sequence involving an initial spin excitation, a square-wave gradient modulation *G*(*t*) (blue boxes), and an echoed background gradient modulation *G*_0_(*t*) (in red in panels a and b) deriving from a spin-echo sequence involving a *π*-pulse. Panel (b) expresses the sequence’s modulations in terms of square-wave functions, and the delay x = *TE*/*N* in this particular example is assumed to be *N* = 6. NMR/MRI acquisitions are performed at the end of the sequence. (**c**) Normalized spin signal expected as a function of the total evolution time *TE*, describing the overall diffusion-driven attenuation in terms of different factors *β*(*TE*) that relate to first- and second-order descriptions of the background gradient distribution *G*_0_, and of an externally applied gradient *G* (see text). The different signs reflect the attenuations expected when *G* acts parallel or antiparallel to the background gradient’s direction. All plots assumed $\langle {G}_{0}\rangle /G=0.1$, $\sqrt{\langle {\rm{\Delta }}{G}_{0}^{2}\rangle }/\langle {G}_{0}\rangle =1$, and *N* = 6.

The first one reflects the diffusion weighting due to the applied external gradient; the second arises purely from the internal gradient; and the last term is a cross-product reflecting the interference between ${\overrightarrow{G}}_{0}$ and $\overrightarrow{G}$. As typically *G* ≫ *G*_0_ the second term, arising from the spins’ diffusion in the background gradient, will in general be negligible. By contrast, the ${\beta }_{\overrightarrow{G}\cdot {\overrightarrow{G}}_{0}}\propto G\,{G}_{0}$ cross-term can facilitate the detection of *G*_0_-driven effects, as these will appear amplified by the applied gradient strength *G*. To visualize how this measurement could be done consider the simplest scenario of a free, unrestricted diffusion taking place in the presence of a susceptibility-induced background gradient. Figure 1a,b show a gradient modulation scheme that, based on a Carr-Purcell Meiboom-Gill (CPMG) type modulation^51, 52^ with delay *x* = *TE*/*N*, could then enable one to measure this cross-term effect. Assuming for simplicity that all other sources of decoherence have been normalized out, the magnetization’s attenuation for the sequence in Fig. 1a will be defined by the three terms in Eq. (2). At an echo time *TE*, these are

3 $${\beta }_{{G}^{2}}(TE)=\frac{1}{12}{\gamma }^{2}{G}^{2}{D}_{0}\frac{T{E}^{3}}{{N}^{2}},$$

4 $${\beta }_{{G}_{0}^{2}}(TE)=\frac{1}{12}{\gamma }^{2}{G}_{0}^{2}{D}_{0}T{E}^{3},$$

5 $${\beta }_{\overrightarrow{G}\cdot {\overrightarrow{G}}_{0}}(TE)=\frac{1}{4}{\gamma }^{2}\overrightarrow{G}\cdot {\overrightarrow{G}}_{0}{D}_{0}\frac{T{E}^{3}}{{N}^{2}},$$

where *D*_0_ is the free diffusion coefficient. The first term is the dominant weighting, while the second term (Equation (4)) will be neglected as it is a pure background-gradient effect. The information being here sought is thus contained in the cross-term, Eq. (5).

Considering that the signal detected in NMR or MRI experiments will nearly invariably arise from an ensemble of spins feeling different background gradients, the univocal determination of ${\overrightarrow{G}}_{0}$ within a bulk sample or voxel becomes unrealistic. Instead, we aim to extract the parameters that characterize these gradient distributions, in terms of a model which we shall base on a perturbative statistical description of ${\overrightarrow{G}}_{0}$’s distribution. To this end we assume that the background gradients within each pixel are described by a Gaussian random distribution of average value $\langle {\overrightarrow{G}}_{0}\rangle$ and of variance $\langle {({\overrightarrow{G}}_{0}-\langle {\overrightarrow{G}}_{0}\rangle )}^{2}\rangle$. This model is also a good representation, up to a second order approximation, for other potential distributions. Using the property of a Gaussian random variable *X*, $\langle {e}^{X}\rangle ={e}^{\langle X\rangle }+{e}^{\langle {(X-\langle X\rangle )}^{2}\rangle /2}$, the cross-term in Eq. (5) becomes

6 $${\beta }_{\overrightarrow{G}\cdot {\overrightarrow{G}}_{0}}(TE)=\frac{1}{4}{\gamma }^{2}{D}_{0}\frac{T{E}^{3}}{{N}^{2}}\,\overrightarrow{G}\cdot \langle {\overrightarrow{G}}_{0}\rangle +\frac{1}{32}{\gamma }^{4}{D}_{0}^{2}\frac{T{E}^{6}}{{N}^{4}}\,\overrightarrow{G}\cdot \langle {\rm{\Delta }}{\overrightarrow{G}}_{0}{\rm{\Delta }}{\overrightarrow{G}}_{0}\rangle \cdot \overrightarrow{G},$$

where the first order term depends of the average of the background gradient distribution $\langle {\overrightarrow{G}}_{0}\rangle$, and the second order term is defined by the orientation-dependent variance of the distribution: ${\rm{\Delta }}{\overrightarrow{G}}_{0}={\overrightarrow{G}}_{0}-\langle {\overrightarrow{G}}_{0}\rangle$. This variance originates what we denominate the internal gradient-distribution tensor (IGDT) $\langle {\rm{\Delta }}{\overrightarrow{G}}_{0}{\rm{\Delta }}{\overrightarrow{G}}_{0}\rangle$, a dyadic product that can be traced to the diffusion-driven physical processes that originate its observable effects, and which stem from the quadratic form that the attenuation of an NMR signal takes vis-a-vis the intervening gradients, Eq. (1).

Figure 1c examines, for an isotropic diffusion case, the contributions that these different attenuation factors will have to the overall signal. The black solid line is the signal decay given solely by the applied gradient term and by (the much weaker) background gradient diffusion term: ${\beta }_{{G}^{2}}(TE)+{\beta }_{{G}_{0}^{2}}(TE)$ in Eqs (3) and (4). Notice that these contributions are independent of the applied gradient direction. By contrast, depending on whether $\overrightarrow{G}$ is parallel or antiparallel to $\langle {\overrightarrow{G}}_{0}\rangle$, the first-order diffusion-weighting term of ${\beta }_{\overrightarrow{G}\cdot {\overrightarrow{G}}_{0}}$ in Eq. (6) will add or subtract to the signal decay (orange curves in Fig. 1c). Conversely, the IGDT weighting given by the second order term of Eq. (6), will always be positive and increase the signal attenuation (dashed blue line). These different effects of these various terms to the overall signal attenuation can be exploited to distinguish among them. As explained in the next paragraph, a particular useful tool for doing so rests on the different symmetries of the gradient and spin-echo modulations involved in the sequence shown in Fig. 1a,b. It is also worth noticing that although Eq. (6) was derived on the basis of an isotropic diffusion, Supporting Information 1 generalizes this result to restricted anisotropic diffusion cases which we consider from now on.

### How to measure IGDTs

**Figure 2:**
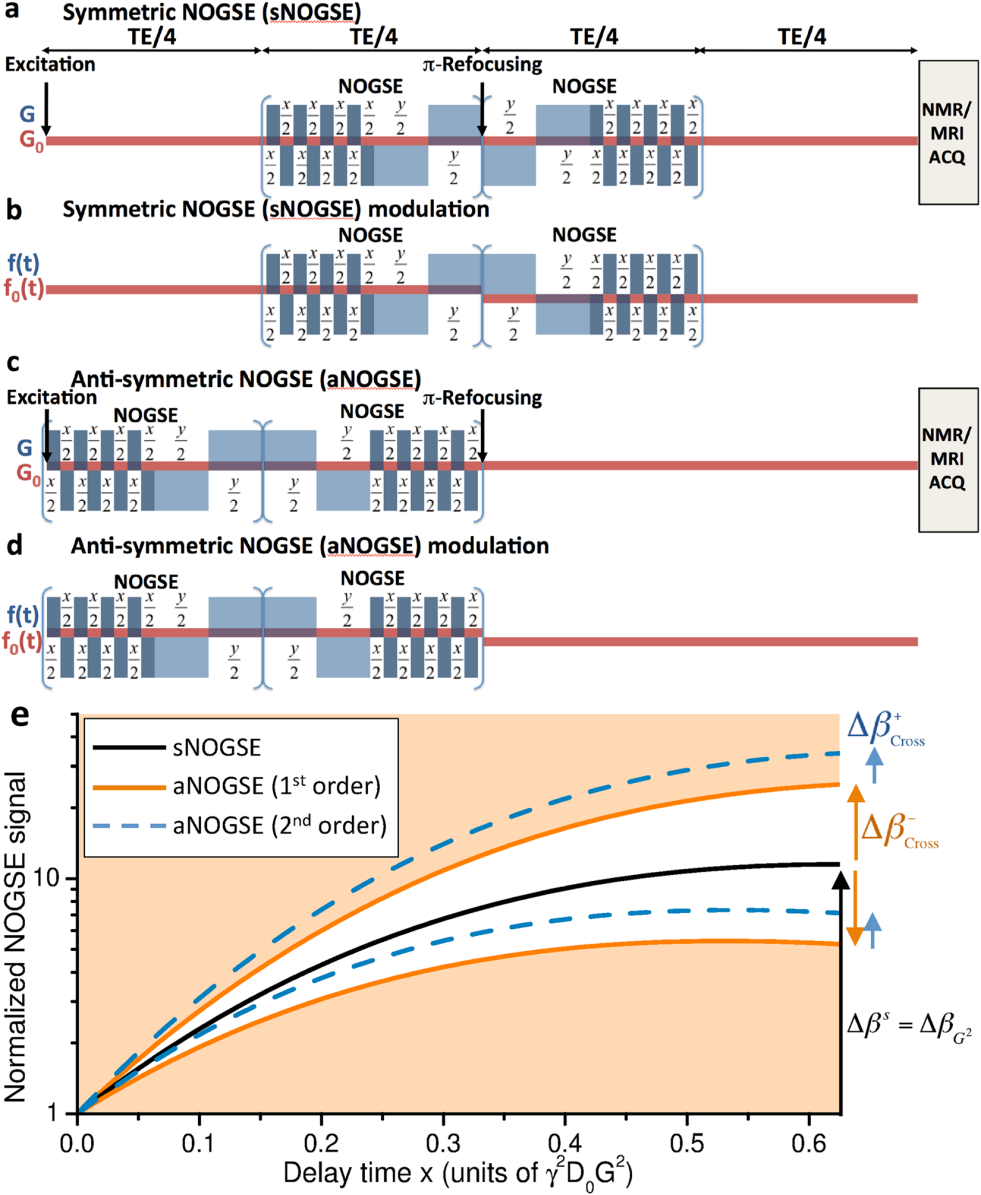
Selective sensing of averages and variances arising from the gradient distributions by (**a**) symmetric-NOGSE (sNOGSE) and (**c**) asymmetric-NOGSE (aNOGSE) sequences. The corresponding modulation waveforms *f*(*t*) and *f*_0_(*t*) are shown in panel (**b**) and (**d**) for sNOGSE and aNOGSE respectively. (**e**) Normalized NOGSE signals expected as a function of the delay *x*, where $(N-\mathrm{2)}x+2y=T{E}_{NOGSE}=TE/2$. By normalizing signals for *x* ≪ *y* and then changing *x*, *y* while keeping all other parameters – including *N* and *TE*– constant, s- and a-NOGSE sequences enable the characterization of the IGDT effects. sNOGSE’ s waveform is symmetric vs the central *π* refocusing pulse; all cross-terms with the internal gradients are thus zero, freeing the experiment from all internal gradients effects (panel c, black solid line). By contrast, the aNOGSE’ s waveform will be affected by both the 1^st^ and 2^nd^ order effects related to the internal gradient cross-terms. The legends describes the different weightings of these attenuation factors stemming from the background gradient distributions. The quantities ${\rm{\Delta }}{\beta }_{Cross}^{-}$ and ${\rm{\Delta }}{\beta }_{Cross}^{+}$ used to selectively determine the average $\langle {G}_{0}\rangle$ and variance $\langle {({\rm{\Delta }}{G}_{0})}^{2}\rangle$ are shown with arrows, where the different signs denote the application of the external gradient *G* parallel and anti-parallel to the background gradient direction. Other assumptions include $\langle {G}_{0}\rangle /G=0.1$, $\sqrt{\langle {\rm{\Delta }}{G}_{0}^{2}\rangle }/\langle {G}_{0}\rangle =1$ and *N* = 8.

The above treatment discusses the influence of IGDTs on NMR signal decays as a function of *TE*, decay whose measurement would be affected by other sources of decoherence like *T*_2_. Alternatives involving changing the number of gradient oscillations, of refocusing *π*-pulses or the gradient intensities, would also be compromised by instrumental sources of error (pulse imperfections, eddy currents, etc.) that could mask the subtle effects being sought. We propose instead a method that exploits the timing symmetries of sequences involving a constant number of gradient oscillations, equal gradient intensities and constant number of pulses, based on non-uniform oscillatory gradient spin-echo (NOGSE) waveforms^20, 21, 44^. As explained elsewhere, these sequences are designed so that all potential sources of signal decay that are unrelated to diffusivity – particularly those arising from *T*_2_ and ${T}_{2}^{\ast }$ effects as well as from pulse and gradient imperfections– are factored out. Still, this would leave the problem of how to distinguish among various diffusion-driven effects, and in particular how to differentiate among the three sources of signal decay introduced in Fig. 1c. To this end Fig. 2a–d propose the use of two NOGSE sequence variants, possessing equal gradient and pulse modulation blocks but different symmetries. These different symmetries will allow us to distinguish the effects of the 2^nd^ order IGDT-derived changes illustrated in Fig. 1c, from those arising from the individual background and applied gradient terms as well as from their 1^st^-order cross-term. To visualize how the two NOGSE variants in Fig. 2a–d introduce these distinctions, we revisit the different ways by which the spin- and the gradient-echo components in them, will modulate the relative contributions of the various attenuation terms in Eq. (2). The first two contributions

7 $${\beta }_{{G}^{2}}(TE)=\tfrac{{\gamma }^{2}{G}^{2}}{2}{\int }_{-\infty }^{\infty }d\omega \frac{{D}_{G}(\omega )}{{\omega }^{2}}{|F(\omega ,TE)|}^{2}$$

and

8 $${\beta }_{{G}_{0}^{2}}(TE,{\overrightarrow{G}}_{0})=\tfrac{{\gamma }^{2}{G}_{0}^{2}}{2}{\int }_{-\infty }^{\infty }d\omega \frac{{D}_{{G}_{0}}(\omega )}{{\omega }^{2}}{|{F}_{0}(\omega ,TE)|}^{2},$$

with ${D}_{G}(\omega )=[{\overrightarrow{G}}^{\dagger }\cdot {\bf{D}}(\omega )\cdot \overrightarrow{G}]/{G}^{2}$ and ${D}_{{G}_{0}}(\omega )=[{{\overrightarrow{G}}_{0}}^{\dagger }\cdot {\bf{D}}(\omega )\cdot {\overrightarrow{G}}_{0}]/{G}_{0}^{2}$, depend solely on the diffusion spectral densities projected by the external and internal gradient modulation functions respectively. As such, the attenuations coming from the first two terms in Eq. (2) will be independent of the timing symmetry of the sequence. By contrast, the attenuation deriving from the cross term

9 $${\beta }_{\overrightarrow{G}\cdot {\overrightarrow{G}}_{0}}(TE)=\overrightarrow{G}\cdot [{\int }_{-\infty }^{\infty }d\omega \,2\,{\rm{Re}}\{{F}^{\dagger }(\omega ,TE)\frac{{\bf{D}}(\omega )}{{\omega }^{2}}{F}_{0}(\omega ,TE)\}]\cdot {\overrightarrow{G}}_{0}$$

will depend on the product of the filter functions ${F}^{\dagger }(\omega ,TE)$ and *F*_0_(*ω*, *TE*) associated to the external and internal gradients. Notice moreover that **D**(*ω*)/*ω* ^2^ is symmetric vs frequency; therefore the integrand in Eq. (9) may be an even or an odd function, depending on the symmetry of the *f*(*t*) and *f*_0_(*t*) modulating functions. In the NOGSE sequences of Fig. 2 *f*_0_(*t*) is always odd, meaning that the symmetric (sNOGSE) waveform, comprising an external gradient modulation that is mirrored with respect to a central refocusing *π*-pulse, will lead to an odd total integrand in Eq. (9). By contrast the asymmetric (aNOGSE) sequence, where the gradient waveform is placed entirely on one half of the sequence, will lead to an even modulation. It follows that the ${\beta }_{\overrightarrow{G}\cdot {\overrightarrow{G}}_{0}}$ term will be identically zero in sNOGSE, but will be non-null in aNOGSE (see Supplements 2 and 3 for a more detailed description of these arguments). Since all other possible sources of decoherence – including pulse and gradient imperfections, ${T}_{2}^{\ast }$, *T*_2_ and both the *G*- and *G*_0_-derived gradient diffusion weightings– remain identical in both sNOGSE and aNOGSE^20, 21, 44^, one can selectively sense the internal gradients cross-terms while removing all these other sources of decoherence by a suitable analysis, as follows.

Obtaining from ${\beta }_{\overrightarrow{G}\cdot {\overrightarrow{G}}_{0}}(TE)$-derived cross-terms the desired IGDTs, requires measuring the NMR signal decays for sNOGSE and aNOGSE sequences as a function of *x* -values, ranging from *x* = *y* (i.e., a CPMG-like gradient modulation) to x ≪ *y* (basically a single-echo gradient modulation). Normalizing the CPMG-modulated signals by the single-echo modulated ones^20, 21, 44^ factors out all non-diffusion sources of decoherence, leading to attenuation curves like those depicted in Fig. 2e. These normalized ${M}_{CPMG}(TE)/{M}_{Single-echo}(TE)=\exp (-{\rm{\Delta }}\beta )$ amplitude modulations depend on an attenuation factor Δ*β*, which will be ${\rm{\Delta }}{\beta }^{s}={\rm{\Delta }}{\beta }_{{G}^{2}}$ for the symmetric-NOGSE modulation, and ${\rm{\Delta }}{\beta }^{a}={\rm{\Delta }}{\beta }_{{G}^{2}}+{\rm{\Delta }}{\beta }_{\overrightarrow{G}\cdot {\overrightarrow{G}}_{0}}$ for the aNOGSE one. Subtracting Δ*β* ^*a*^ and Δ*β* ^*s*^ for every *TE* leads to a pure $\Delta {\beta }_{\overrightarrow{G}\cdot {\overrightarrow{G}}_{0}}$ cross-term contribution, from which the mean- and the variance-contributions (Equation (6)) can be distinguished by repeating the measurements for positive and negative gradients, $\pm \overrightarrow{G}$. In the resulting experiments the ${\rm{\Delta }}{\beta }_{\pm \overrightarrow{G}\cdot {\overrightarrow{G}}_{0}}$ contributions will have a first-order cross-term that is linear in $\overrightarrow{G}$ and thus have opposite signs, whereas the second-order cross-term is quadratic in $\overrightarrow{G}$ and thus remains constant. Therefore, subtracting and adding these contributions, allows one to obtain the average (1st order term) and the variance (2nd order) terms, respectively: ${\rm{\Delta }}{\beta }_{{\rm{Cross}}}^{-}={\rm{\Delta }}{\beta }_{+\overrightarrow{G}\cdot {\overrightarrow{G}}_{0}}-{\rm{\Delta }}{\beta }_{-\overrightarrow{G}\cdot {\overrightarrow{G}}_{0}}\propto \overrightarrow{G}\cdot \langle {\overrightarrow{G}}_{0}\rangle$ and ${\rm{\Delta }}{\beta }_{{\rm{Cross}}}^{+}={\rm{\Delta }}{\beta }_{+\overrightarrow{G}\cdot {\overrightarrow{G}}_{0}}+{\rm{\Delta }}{\beta }_{-\overrightarrow{G}\cdot {\overrightarrow{G}}_{0}}\propto \overrightarrow{G}\cdot \langle {\rm{\Delta }}{\overrightarrow{G}}_{0}{\rm{\Delta }}{\overrightarrow{G}}_{0}\rangle \cdot \overrightarrow{G}$. Finally, applying the gradient $\overrightarrow{G}$ on several suitable directions^7, 8^ allows one to reconstruct both the internal gradient average vector $\langle {\overrightarrow{G}}_{0}\rangle$ and the second-rank IGDT tensor $\langle {\rm{\Delta }}{\overrightarrow{G}}_{0}{\rm{\Delta }}{\overrightarrow{G}}_{0}\rangle$. Notice that all these considerations hold for the case of an isotropic diffusion, where **D**(*ω*) is represented by a scalar function which can be factored out of the calculations. If anisotropic diffusion effects are present the diffusion tensor **D**(*ω*) needs to be reconstructed first, and then used to revisit the full analysis based on Eq. (9). This is not overtly onerous, as **D**(*ω*) can be extracted based on measured sNOGSE amplitude modulation Δ*β* ^*s*^, which is insensitive to background gradients and only relies on $\overrightarrow{G}$ to encode the microscopic apparent diffusion coefficient. If this measurement is done over a set of suitable gradient directions, information akin to that arising in diffusion tensor imaging (DTI) emerges. Alternatively, one can normalize ${\rm{\Delta }}{\beta }_{{\rm{Cross}}}^{\pm }$ by Δ*β* ^*s*^, to map the anisotropy-imposed IGDT and the average background gradient without influence from – or knowledge of– the anisotropic diffusion weighting.

### Experimentally mapping IGDTs in free and constrained diffusion instances

**Figure 3:**
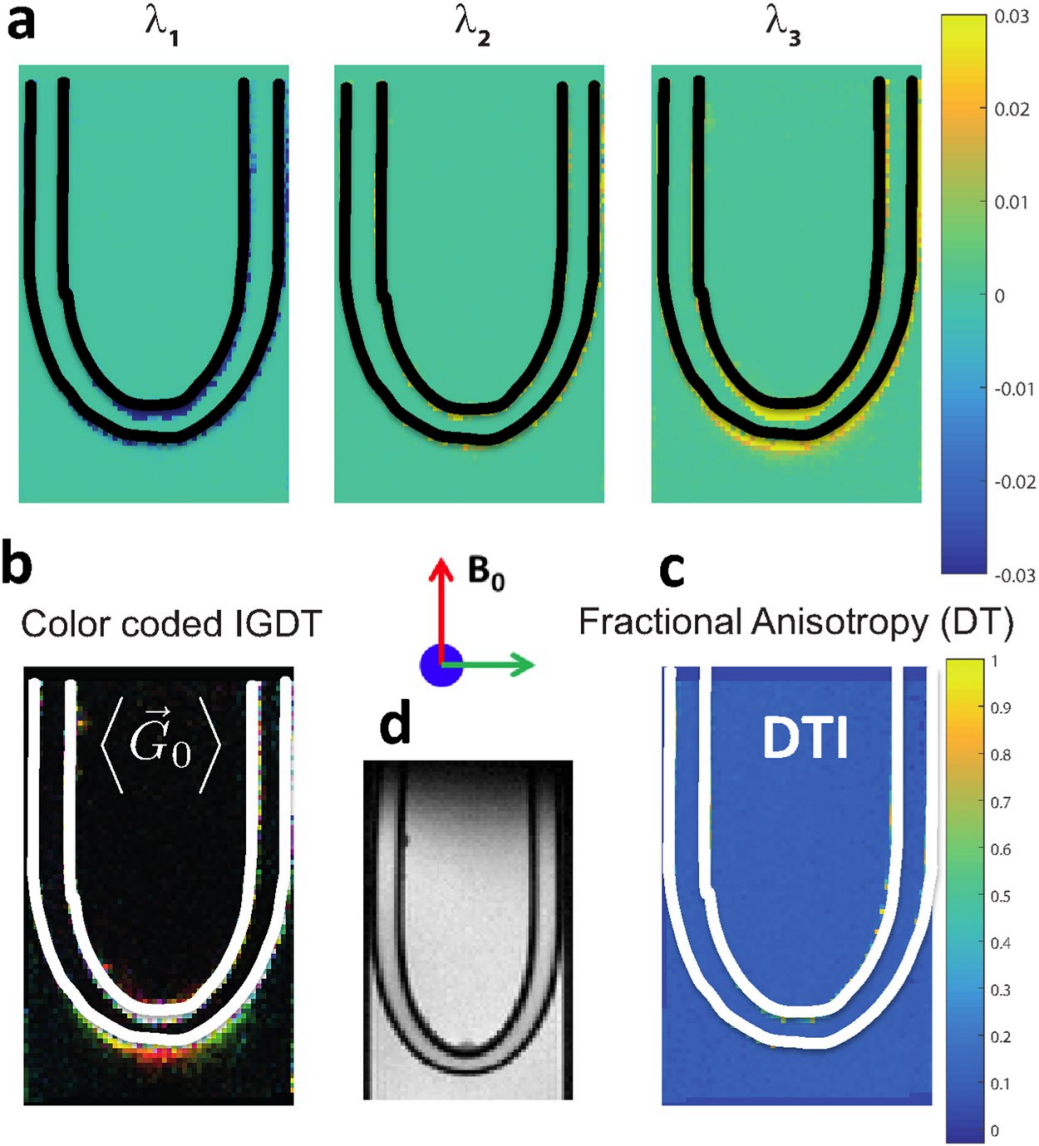
Mapping the average background gradient in a water phantom incorporating a rubber “U-tube”, also filled with water (see panel (**d**) for a magnetic resonance image of the analyzed object). (**a**) Images of the eigenvalues determined for the IGDT using six non-collinear $\overrightarrow{G}$ directions [1, 1, 0], [−1, 1, 0], [0, 1, −1], [0, −1, −1], [1, 0, −1], [−1, 0, −1]. A black mask was applied on the position occupied by the tube. Since the sample is relatively uniform most eigenvalues are null; only in the proximity of the “U” bend do spins feel the susceptibility distortions, originating the non-null eigenvalues *λ*_3_ appearing yellow in the proximity of the bend. Other eigenvalues are indistinguishable from 0; *λ*_3_, the eigenvector with the largest eigenvalue, is therefore in essence a vector that defines the orientation of the average background gradient. (**b**) Color-coded orientation maps arising from this average background gradient $\langle {G}_{0}\rangle$ [red: z-axis (up-down), blue: x-axis (in-out), green: y-axis (left-right)]. Parameters for these NOGSE MRI measurements were: *N* = 8, *G* = 21 G/cm, *TE*_*NOGSE*_ = 10 ms; spatial resolution =125 × 125 × 1000 (μm)^3^; repetition and echo times *TR*/*TE* = 500/28 ms, number of averages NA = 8. (**c**) The DTI tensor determined from the sNOGSE amplitude modulation Δ*β* ^*S*^ was used to calculate and plot its fractional anisotropy ${\rm{FA}}=\sqrt{\frac{3}{2}}\frac{\sqrt{{({\lambda }_{1}-\bar{\lambda })}^{2}+{({\lambda }_{2}-\bar{\lambda })}^{2}+{({\lambda }_{3}-\bar{\lambda })}^{2}}}{\bar{\lambda }}$ where $\bar{\lambda }=\sqrt{{\lambda }_{1}^{2}+{\lambda }_{2}^{2}+{\lambda }_{3}^{2}}$ and *λ*_*i*_ are the three DTI eigenvalues, showing no information as diffusion is in this instance isotropic in all positions. A white mask was also applied in the latter two panels. An EPI acquisition sequence^1^ was used for the all images; the typical SNR was >100 at its lowest. A full set of measurements took <2 min to acquire.

**Figure 4:**
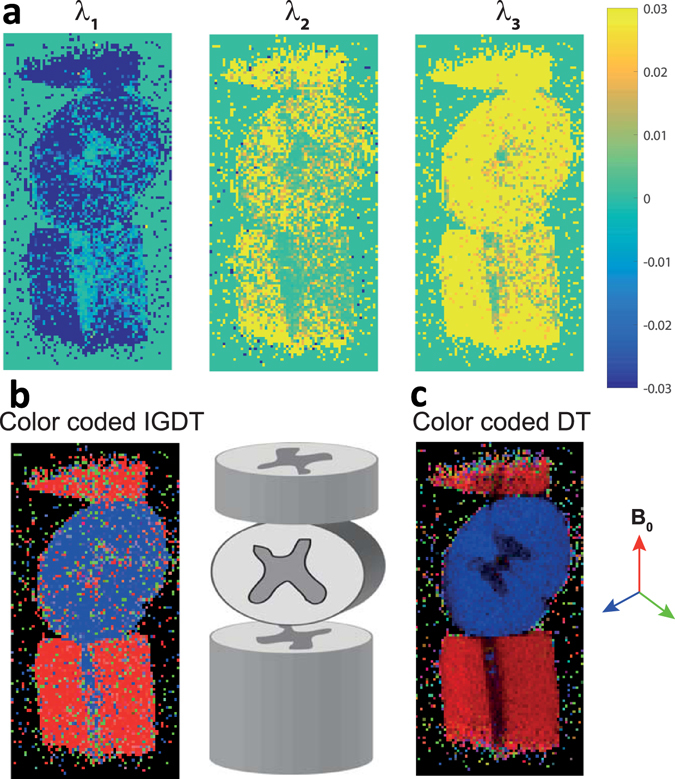
Mapping IGDT in biological tissues. (**a**) IGDT eigenvalues observed for a spinal cord specimen, examined in a 10 mm NMR tube filled with Fluorinert^®^ (cartoon in center exemplifies this model phantom). (**b**) Color-coded orientation maps generated from the directions of the first eigenvector (the one with lowest eigenvalue) with respect to the main magnetic field [red: z-axis (up-down), blue: x-axis (in-out), green: y-axis (left-right)]. The vector magnitude was weighted with a fractional anisotropy given by ${\rm{FA}}=\sqrt{\frac{3}{2}}\frac{\sqrt{{({\lambda }_{1}-\bar{\lambda })}^{2}+{({\lambda }_{2}-\bar{\lambda })}^{2}+{({\lambda }_{3}-\bar{\lambda })}^{2}}}{\bar{\lambda }}$ to highlight its orientation, where $\bar{\lambda }=\sqrt{{\lambda }_{1}^{2}+{\lambda }_{2}^{2}+{\lambda }_{3}^{2}}$ and *λ*_*i*_ are the three IGDT eigenvalues. Parameters for the NOGSE MRI measurements were: *TR*/*TE* = 4000/50 ms, resolution = 156 × 156 × 1000 μm^3^, six pairs of opposing-gradient NOGSE encodings according to the orientations given in Fig. 3, NA = 4, *G* = 35 G/cm, total number of NOGSE oscillations of ten, total NOGSE gradient modulation time =20 ms. A *T*_2_~50–60 ms was measured in these white matter experiments, and the shortest delay *x* was 140 *μ*s. (**c**) Microscopic DTI tensor determined from the sNOGSE amplitude modulation Δ*β* ^*S*^ is shown for comparison to demonstrate the consistency of the orientations. EPI sequences were used for collecting all images, the typical SNR was >35 at its lowest. A full set of measurements took 13 minutes to complete.

As proof-of-principle demonstrations of the concepts just derived, a series of MRI experiments were carried out on model systems. A simple “U-tube” (Fig. 3) was chosen as a case where internal gradient-distribution anisotropies related to morphology show up, yet they fall beyond the realm of DTI characterization capabilities due to involving an unrestricted, isotropic diffusion case. Three fixed pig spinal cord sections stacked on top of one another and oriented parallel and perpendicular to *B*_0_ (Fig. 4) were chosen as a case where IGDT anisotropies were accompanied by a highly anisotropic diffusion, that allowed us to contrast our results with those of a DTI procedure. To study these systems sNOGSE and aNOGSE experiments were performed on a 9.4 T vertical bore Bruker AVANCE III, equipped with gradients capable of producing 291 G/cm in all directions. The samples were fitted within a 10 mm NMR tube filled with water for the “U-tube” and with Fluorinert^®^ for the spinal cords. Two sets of NOGSE images – one with *x* ≪ *y* and one with *x* = *y*– were recorded using the sNOGSE and aNOGSE sequences introduced in Fig. 2; the signals’ amplitude modulations Δ*β* were then measured for 6 non-collinear directions {[1, 1, 0], [−1, 1, 0], [0, 1, −1], [0, −1, −1], [1, 0, −1], [−1, 0, −1]}, and the resulting data diagonalized to obtain the IGDT’ s eigenvalues and eigendirections.

In the “U tube” case, the internal gradient distribution affecting most pixels in the image is not significant; the sole major field distortions arise due to the macroscopic bending of an object inside the sample and magnet. Furthermore there are no restrictions to diffusion, and so this constitutes a suitable case for determining IGDT’s abilities to map orientations based on measuring the average background gradient vector per pixel. To analyze these data we relied on the fact that ${\rm{\Delta }}{\beta }^{a}-{\rm{\Delta }}{\beta }^{s}={\rm{\Delta }}{\beta }_{\overrightarrow{G}\cdot {\overrightarrow{G}}_{0}}\propto \overrightarrow{G}\cdot \langle {\overrightarrow{G}}_{0}\rangle$. From 6 non-collinear directions of an applied external gradient, a tensor could be reconstructed, which in this case was just a vector with only one non-null eigenvalue (Fig. 3a). The orientation of the corresponding eigenvector corresponds in essence to the orientation of the background gradient vector $\langle {\overrightarrow{G}}_{0}\rangle$ (Fig. 3b). This vector is null everywhere except in the proximity of the “U”-tube’s bend, where it becomes transversal to the tube’s orientation. This serves as demonstration of how internal gradients can be mapped by NOGSE sequences, to extract orientations in cases of unrestricted or isotropic diffusion – instances where DTI cannot give any information (Fig. 3c). It also constitutes an example of how these methods can extract morphologies and anisotropic features in mm (i.e., on supra-diffusional) length-scales, without requiring sample reorientations within the magnet.

By contrast, gradient distributions for the spinal cords case are strong, and their intravoxel dependence can no longer be neglected. Therefore, in line with what was previously described, sNOGSE and aNOGSE images for *x* ≪ *y* and *x* = *y* cases were measured for the six pairs of non-collinear gradient directions mentioned earlier, {±[1, 1, 0], ±[−1, 1, 0], ±[0, 1, −1], ±[0, −1, −1], ±[1, 0, −1], ±[−1, 0, −1]}. Every pixel of the images arising from these experiments was then manipulated as explained earlier, to determine $\Delta {\beta }_{Cross}^{+}={\rm{\Delta }}{\beta }_{+\overrightarrow{G}\cdot {\overrightarrow{G}}_{0}}+{\rm{\Delta }}{\beta }_{-\overrightarrow{G}\cdot {\overrightarrow{G}}_{0}}$ normalized with Δ*β* ^*s*^ to remove the anisotropic diffusion weighting. From this the IGDTs were determined, taking into consideration the anisotropic nature of the diffusion that was probing them (see Supporting Information 2 for further details). To translate the IGDT’s eigenvalues and eigenvectors into local fiber orientations, it was assumed that multiple longitudinal and parallel fibers are oriented along the principal axis of the spinal cord. The ensuing IGDT will have a minimal principal eigenvalue (minimal gradient-distribution variance) along the principal axes of these cylindrical anisotropic structures, and therefore the eigenvector corresponding to the smallest of all eigenvalues, will reveal the fibers’ orientation^43^. Figure 4a shows the three eigenvalues arising from the analysis, which give the variances of the gradient distributions along the three principal axes of the IGDT. These clearly demonstrate the anisotropy of the internal gradient distributions. The largest of these eigenvalues, *λ*_3_, represents the maximum distribution width of the gradients; as could be expected from the variation of magnetic fields in packed cylinders, it is much larger than the other two eigenvalues^43^. A fiber orientation mapping can then be built using the orientation of the IGDT eigenvector possessing the minimal eigenvalue; Fig. 4b shows this fiber orientation map, using a RGB color-mapping scheme [red: z-axis (up-down), blue: x-axis (in-out), green: y-axis (left-right)]. Clearly, the white matter of the lower and upper spinal cords segment point parallel to *B*_0_ (red in our RGB scheme), whereas the middle spinal cord section points perpendicular to the main field (blue in our RGB scheme). As explained earlier, the amplitude modulations Δ*β* ^*s*^ provided by sNOGSE measurements, can also be used to extract the microscopic apparent diffusion tensor. This DTI information should lead to a similar information on the fibers’ orientation as that which becomes available from IGDTs, something that is well corroborated by Fig. 4c. This excellent agreement between the IGDT and the microscopic DTI orientations supports the validity of the IGDT approach. The stronger contrast to noise evidenced by the DTI images likely reflects the relatively small susceptibility-induced internal gradients arising in white matter.

## Discussions

This report provides a new approach for mapping susceptibility-induced internal gradients – and from these, morphologies at a variety of length scales– even in the absence of restrictions in molecular diffusivity. To this effect a novel internal gradient-distribution tensor concept is put forward, and sufficiently accurate techniques to measure it, are devised. The outcome of this analysis is an alternative that complements well-established DTI methods for mapping orientations and morphologies. The experiments developed to perform these measurements rely, like DTI, on translational diffusivity, but these are only exploited as probes of the internal gradients. Hence, unlike DTI measurements, IGDTs do not demand the presence of restricted diffusion effects. The approach is also distinctly different from other methods such as susceptibility tensor imaging (STI) and/or ${T}_{2}^{\ast }$ mapping, as it fully benefits from highly spin-echoed sequences that provide robustness towards global *B*_0_ distortions, while requiring no rotation of the sample in the magnet to deliver orientational insight. The measurement of IGDTs could therefore become an important approach to complement anisotropic scenarios where DTI effects are tenuous, i.e., where diffusion path lengths do not extend far enough to probe a pore’s boundary constraint. In particular, IGDT determinations in high-field scenarios could allow one to measure oriented morphologies whose diameters exceed ≈50–100 *μ*m filling the gap of resolutions between conventional imaging (>100 *μ*m) and DTI-based imaging (<50 μm). This method could also show important benefits in non-invasive studies using ultra-high field MRI of structured systems, including studies of porous media and living tissues, where *B*_0_-magnified internal gradients could serve as fingerprints of a system’ s composition and structure. The success of these measurements will naturally depend on the strength of the background gradients and on their gradient-distribution variances; i.e., they will strongly depend on the nature of the sample of interest. The approach’s success will also depend on ensuring that the relative change of the diffusion-weighting attenuation factors arising due to the cross-term interference between the applied and background gradient, are larger than the inverse of the signal-to-noise-ratio of the spin signal. This need for the changes being sought to exceed the experimental uncertainties, will in turn define the optimal magnetic field strength and/or external gradient that should be used to best exploit this methodology; in principle it would appear that higher fields would invariable help to define the observable, yet further research is needed to assess this. Further interesting options arise if spin-diffusion^53^ – as opposed to physical diffusion– is used as probing mechanism; in such instances much finer decoherence phenomena such as those arising from anisotropic susceptibilities, could become observable for the first time.

## Electronic supplementary material

Supporting Information
