# Supplementary material for: Internal gradient distributions: A susceptibility-derived tensor delivering morphologies by magnetic resonance
Source: Sci Rep. 2017 Jun 12;7:3311. doi: 10.1038/s41598-017-03277-9 (PMC5468317; doi:10.1038/s41598-017-03277-9)
Supplement: Supplementary file 1 — Supporting Information [file 41598_2017_3277_MOESM1_ESM.pdf]

Supporting Information

Internal gradient distributions: A susceptibility-derived tensor delivering morphologies by magnetic resonance

Gonzalo A. Álvarez, Noam Shemesh, and Lucio Frydman

Supporting Information 1: A formal derivation of the internal gradient distribution tensor

The normalized magnetization arising from an ensemble of non-interacting and equivalent spins under the effects of a sequence of pulses or modulating gradients is $M(t) = \left\langle e^{-i\phi(t)} \right\rangle$, where the brackets account for an ensemble average over the random phases $\phi(t)$. For the spin-echo sequences being considered in this work, the average phase $\langle \phi(t) \rangle$ will be equal to zero. Assuming that the random phase $\phi(t)$ has a Gaussian distribution [48], $M(t) = \exp\left\{-\frac{1}{2}\left\langle \phi^2(t) \right\rangle\right\}$, the signal will evidence a decay depending on the attenuation factor $\beta(t) = \frac{1}{2}\left\langle \phi^2(t) \right\rangle$. With most sources of decoherence normalized out by the constant-time, constant-pulses-number, fixed-number-of-gradients nature of the NOGSE sequences assayed [20, 21, 44], we ascribe to diffusion effects as the sole source of this attenuation. It is then convenient to describe the β-factor in terms of the gradient modulating function $\vec{G}_{\mathrm{tot}}(t')$ [45–47]:

$$\beta(TE) = \frac{\gamma^2}{2} \int_0^{TE} dt' \int_0^{TE} dt'' \vec{G}^{\dagger}_{\mathrm{tot}}(t') \cdot \langle \vec{r}(t') \vec{r}(t'') \rangle \cdot \vec{G}_{\mathrm{tot}}(t'') \qquad \text{(S.1)}$$

$$= \frac{\gamma^2}{2} \int_{-\infty}^{\infty} dt' \int_{-\infty}^{\infty} dt'' \vec{G}^{\dagger}_{\mathrm{tot}}(t', TE) \cdot \mathbf{g}\left(t'' - t'\right) \cdot \vec{G}_{\mathrm{tot}}(t'', TE), \qquad \text{(S.2)}$$

where in the second equation we redefined the gradient modulation function such that $\vec{G}_{\mathrm{tot}}(t', TE) = 0$ if $t' < 0$ or $t' > TE$ (i.e., outside the total evolution time range). The evolution is given in terms of a tensorial correlation function reflecting the displacements' fluctuations $\mathbf{g}(\tau) = \langle \Delta\vec{r}(t')\Delta\vec{r}(t' + \tau) \rangle$; i.e. $g_{i,j} = \langle \Delta x_i(t') \Delta x_j(t' + \tau) \rangle$ with i, j representing the spatial axis x, y, z. This correlation function can be related to a diffusion power spectrum $\mathbf{D}(\omega)$ [4, 5, 45, 46] by a Fourier transform: $\mathcal{FT}\left\{\mathbf{g}(\tau)\right\}/\sqrt{2\pi} = \mathbf{D}(\omega)/\omega^2$. In the event of anisotropic diffusion, Eq. (S.1) can thus be recast in its Fourier representation [45–47] as:

$$\beta(TE) = \frac{\gamma^2}{2} \int_{-\infty}^{\infty} d\omega \vec{\mathcal{G}}^{\dagger}_{\mathrm{tot}}(\omega, TE) \cdot \frac{\mathbf{D}(\omega)}{\omega^2} \cdot \vec{\mathcal{G}}_{\mathrm{tot}}(\omega, TE), \qquad \text{(S.3)}$$

where $\vec{\mathcal{G}}_{\text{tot}}(\omega, TE) = \vec{\mathcal{G}}(\omega, TE) + \vec{\mathcal{G}}_0\,(\omega, TE)$, is the filter function introduced in Eq. (1) of the main text.

Considering the applied gradient modulation $\vec{G}(t', TE)$, the internal background gradient modulation $\vec{G}_0(t', TE)$, and their respective filter functions $\vec{\mathcal{G}}(\omega, TE)$ and $\vec{\mathcal{G}}_0\,(\omega, TE)$, the argument of the integral defining this attenuation factor can then be expanded as

$$\begin{aligned}\vec{\mathcal{G}}^{\dagger}_{\text{tot}}(\omega, TE) \cdot \frac{\mathbf{D}(\omega)}{\omega^2} \cdot \vec{\mathcal{G}}_{\text{tot}}(\omega, TE) = &\underbrace{\vec{\mathcal{G}}^{\dagger}(\omega, TE) \cdot \frac{\mathbf{D}(\omega)}{\omega^2} \cdot \vec{\mathcal{G}}(\omega, TE)}_{\text{external gradient dephasing}} \\ &+ \underbrace{\vec{\mathcal{G}}^{\dagger}_0(\omega, TE) \cdot \frac{\mathbf{D}(\omega)}{\omega^2} \cdot \vec{\mathcal{G}}_0(\omega, TE)}_{\text{internal gradient dephasing}} \\ &+ \underbrace{2\Re\left\{\vec{\mathcal{G}}^{\dagger}(\omega, TE) \cdot \frac{\mathbf{D}(\omega)}{\omega^2} \cdot \vec{\mathcal{G}}_0\,(\omega, TE)\right\}}_{\text{cross-term}}.\end{aligned} \tag{S.4}$$

This leads to Eq. (2) of the main text, where $\beta(TE) = \beta_{G^2}(TE) + \beta_{G_0^2}(TE) + \beta_{\vec{G}\cdot\vec{G}_0}(TE)$ and the normalized spin magnetization becomes

$$M(TE) = M_{G^2}(TE) \times M_{G_0^2}\,(TE) \times M_{\vec{G}.\vec{G}_0}(TE). \tag{S.5}$$

Assuming a $\vec{G}(t', TE) = \vec{G}\,f(t', TE)$, involving a strength vector $\vec{G}$ and a time-dependency $f(t', TE)$, then $\vec{\mathcal{G}}(\omega, TE) = \vec{G}\,F(\omega, TE)$ with $F(\omega, TE)$ the Fourier transform of $f(t', TE)$. The applied gradient diffusion attenuation becomes

$$M_{G^2}(TE) = \exp\left\{-\beta_{G^2}(TE)\right\}, \tag{S.6}$$

where

$$\beta_{G^2}(TE) = \tfrac{\gamma^2 G^2}{2} \int_{-\infty}^{\infty} d\omega \frac{D_G(\omega)}{\omega^2}\,|F(\omega, TE)|^2\,, \tag{S.7}$$

and $D_G(\omega) = \left[\vec{G}^{\dagger} \cdot \mathbf{D}(\omega) \cdot \vec{G}\right]/G^2$. Likewise, the pure background gradient decay is independent of the applied gradient

$$M_{G_0^2}\,(TE) = \exp\left\{-\beta_{G_0^2}\,(TE)\right\}, \tag{S.8}$$

where

$$\beta_{G_0^2}(TE,\vec{G}_0) = \tfrac{\gamma^2 G_0^2}{2}\int_{-\infty}^{\infty} d\omega \frac{D_{G_0}(\omega)}{\omega^2}\left|F_0(\omega,TE)\right|^2, \tag{S.9}$$

with $D_{G_0}(\omega) = \left[\vec{G}_0^\dagger \cdot \mathbf{D}(\omega)\cdot \vec{G}_0\right]/G_0^2$, and we have again assumed that $\vec{G}_0(t',TE) = \vec{G}_0\, f_0(t',TE)$ and thereby $\vec{\mathcal{G}}_0(\omega,TE) = \vec{G}_0\, F_0(\omega,TE)$. Finally, the cross-term attenuation will be

$$\beta_{\vec{G}\cdot\vec{G}_0}\left(TE,\vec{G},\vec{G}_0\right) = \tfrac{\gamma^2}{2}\vec{G}^\dagger\cdot\left[\int_{-\infty}^{\infty} d\omega\, 2\mathrm{Re}\left\{F^\dagger(\omega,TE)\,\frac{\mathbf{D}(\omega)}{\omega^2}\,F_0\left(\omega,TE\right)\right\}\right]\cdot\vec{G}_0 = \vec{G}^\dagger\cdot\tilde{\mathbf{D}}\cdot\vec{G}_0, \tag{S.10}$$

where $\tilde{\mathbf{D}} = \frac{\gamma^2}{2}\left[\int_{-\infty}^{\infty} d\omega\, 2\mathrm{Re}\left\{F^\dagger(\omega,TE)\,\frac{\mathbf{D}(\omega)}{\omega^2}\,F_0\left(\omega,TE\right)\right\}\right]$.

Our derivations also assumed that $\vec{G}_0$ can be described by a Gaussian distribution. The cross-term contribution to the attenuation factor turns out to be

$$\beta_{\vec{G}\cdot\vec{G}_0}\left(TE\right) = \vec{G}^\dagger\cdot\tilde{\mathbf{D}}\cdot\left\langle\vec{G}_0\right\rangle + \vec{G}^\dagger\cdot\tilde{\mathbf{D}}\cdot\left\langle\Delta\vec{G}_0\Delta\vec{G}_0\right\rangle\cdot\tilde{\mathbf{D}}\cdot\vec{G}, \tag{S.11}$$

where $\Delta\vec{G}_0 = \vec{G}_0 - \left\langle\vec{G}_0\right\rangle$ and $\left\langle\Delta\vec{G}_0\Delta\vec{G}_0\right\rangle$ is the internal gradient-distribution tensor (IGDT). This second term is always positive, since it is a quadratic term, while the first term depends on the relative sign of the parallel component of $\left[\vec{G}^\dagger\cdot\tilde{\mathbf{D}}\right]_{\|}$ to the background gradient G_0.

For an isotropic diffusion $\mathbf{D}(\omega) = D(\omega)\mathbf{I}$, the attenuation factor get the simplified form

$$\beta_{\vec{G}\cdot\vec{G}_0}\left(TE\right) = \tilde{D}_{\mathrm{iso}}\,\vec{G}^\dagger\cdot\left\langle\vec{G}_0\right\rangle + \tilde{D}_{\mathrm{iso}}^2\,\vec{G}^\dagger\cdot\left\langle\Delta\vec{G}_0\Delta\vec{G}_0\right\rangle\cdot\vec{G}, \tag{S.12}$$

where $\tilde{D}_{\mathrm{iso}} = \frac{\gamma^2}{2}\left[\int_{-\infty}^{\infty} d\omega\, 2\mathrm{Re}\left\{F^\dagger(\omega,TE)\,\frac{D(\omega)}{\omega^2}\,F_0\left(\omega,TE\right)\right\}\right]$.

As an example on the use of this formalism, we consider the sequence of Fig. 1 of the main text and assume free diffusion to derive Eq. (3-5) of the main text. For free diffusion only the tail of the displacement power spectrum $D(\omega)\propto 1/\omega^2$ is important [20, 44]. The purely applied-gradient diffusion term $M_{G^2}(TE)$ is as derived for a CPMG sequence [44]

$$M_{G^2}(TE) = \exp\left\{-\frac{1}{12}\gamma^2 G^2 D_0 \frac{TE^3}{N^2}\right\}, \tag{S.13}$$

where the delay $x = TE/N$. The pure background gradient decay term is in turn the one that corresponds to a spin-echo modulation [44]

$$M_{G_0^2}\left(TE,N\right) = \exp\left\{-\frac{1}{12}\gamma^2 G_0^2 D_0 TE^3\right\}, \tag{S.14}$$

which is independent of x. The cross-term signal-decay contribution is calculated from Eq. (S.10) leading to

$$M_{\vec{G}\cdot\vec{G}_0}(TE) = \exp\left\{\frac{1}{4}\gamma^2\vec{G}\cdot\vec{G}_0 D_0\frac{TE^3}{N^2}\right\}. \tag{S.15}$$

Supporting Information 2: sNOGSE/aNOGSE's: Analytical attenuation expressions for the general case of anisotropic diffusion

We calculate next the normalized spin signal arising from Eq. (S.5),

$$M^{\binom{s}{a}NOGSE}(TE) = M_{G^2}^{\binom{s}{a}NOGSE}(TE) \times M_{G_0^2}(TE) \times M_{\vec{G}.\vec{G}_0}^{\binom{s}{a}NOGSE}(TE), \tag{S.16}$$

for the symmetric and asymmetric non-uniform gradient spin echo modulations ($\binom{\mathrm{s}}{\mathrm{a}}$NOGSE) introduced in Fig. 2. As described in the main text,

$$M_{G^2}^{sNOGSE}(TE) = M_{G^2}^{aNOGSE}(TE) \tag{S.17}$$

as a result of

$$F^{\mathrm{sNOGSE}}(\omega, TE) = F^{\mathrm{aNOGSE}}(\omega, TE) \tag{S.18}$$

in Eq. (S.7). The pure background gradient signal contribution is therefore independent of the applied gradient modulation and direction, providing the same weight for both NOGSE sequences. The cross-term in the attenuation factor for sNOGSE is zero but that for aNOGSE is not, as the products $F^{sNOGSE\dagger}(\omega, TE)\, F_0(\omega, TE)$ and $F^{aNOGSE\dagger}(\omega, TE)\, F_0(\omega, TE)$ in Eq. (S.10) are odd and even functions of ω, respectively. This cross-term between the aNOGSE-modulated applied gradient and the background gradient G_0 will be

$$\beta_{\vec{G}\cdot\vec{G}_0}(TE) = \tfrac{\gamma^2}{2}\vec{G}\cdot\left[\int_{-\infty}^{\infty} d\omega\, 2\mathrm{Re}\left\{\left(F^{aNOGSE}(\omega, TE)\right)^{\dagger}\frac{\mathbf{D}(\omega)}{\omega^2}F_0(\omega, TE)\right\}\right]\cdot\vec{G}_0 = \vec{G}\cdot\tilde{\mathbf{D}}\cdot\vec{G}_0. \tag{S.19}$$

As explained in the main text, the measured spin signal decays for the sNOGSE and aNOGSE sequences as described in Fig. 2e, factor out all non-diffusing sources of decoherence after normalizing them by the single-echo signal [20, 21, 44]. The amplitude of the

NOGSE modulation is then

$$M_{CPMG}(TE)/M_{Single-echo}(TE) = \exp\left(-\Delta\beta\right) = \exp\left[-\left(\beta^{CPMG} - \beta^{Single-echo}\right)\right], \quad \text{(S.20)}$$

where the amplitude contrast of the attenuation factors $\Delta\beta = \beta^{CPMG} - \beta^{Single-echo}$. As the contribution to the attenuation factor that purely depends of the background gradient is independent of the applied gradient modulation its contribution $\Delta\beta_{G_0^2}$ is null, and the amplitude of the attenuation factors is then

$$\Delta\beta = \Delta\beta_{G^2} + \Delta\beta_{\vec{G}\cdot\vec{G}_0}. \quad \text{(S.21)}$$

For the sNOGSE sequence $\Delta\beta^s = \Delta\beta_{G^2}$ as the cross-term is null, and $\Delta\beta^a = \Delta\beta_{G^2} + \Delta\beta_{\vec{G}\cdot\vec{G}_0}$ for the aNOGSE modulation curve. Notice that the contribution of the term that only depends of the applied gradient $\Delta\beta_{G^2}$ is the same for both sequences according to Eqs. (S.17) and (S.18). Then by subtracting $\Delta\beta^a$ and $\Delta\beta^s$, the $\Delta\beta_{\vec{G}\cdot\vec{G}_0}$ cross-term contribution to the amplitude modulation is obtained, where

$$\Delta\beta_{\vec{G}\cdot\vec{G}_0} = \frac{\gamma^2}{2}\vec{G}\cdot\left[\int_{-\infty}^{\infty} d\omega\, 2\mathrm{Re}\left\{\left[F^{aNOGSE}(\omega,TE) - F^{sNOGSE}(\omega,TE)\right]^{\dagger}\frac{\mathbf{D}(\omega)}{\omega^2}F_0\left(\omega,TE\right)\right\}\right]\cdot\vec{G}_0 \quad \text{(S.22)}$$

$$= \vec{G}\cdot\Delta\tilde{\mathbf{D}}\cdot\vec{G}_0, \quad \text{(S.23)}$$

with $\Delta\tilde{\mathbf{D}} = \frac{\gamma^2}{2}\int_{-\infty}^{\infty} d\omega\, 2\mathrm{Re}\left\{\left[F^{aNOGSE}(\omega,TE) - F^{sNOGSE}(\omega,TE)\right]^{\dagger}\frac{\mathbf{D}(\omega)}{\omega^2}F_0\left(\omega,TE\right)\right\}$. Assuming as before a Gaussian distribution for G_0,

$$\Delta\beta_{\vec{G}\cdot\vec{G}_0} = \vec{G}\cdot\Delta\tilde{\mathbf{D}}\cdot\left\langle\vec{G}_0\right\rangle + \vec{G}\cdot\Delta\tilde{\mathbf{D}}\cdot\left\langle\Delta\vec{G}_0\Delta\vec{G}_0\right\rangle\cdot\Delta\tilde{\mathbf{D}}\cdot\vec{G}. \quad \text{(S.24)}$$

where the first term depends on the relative sign of the parallel component of $\left[\vec{G}\cdot\mathbf{D}(\omega)\right]_{\parallel}$ to the background gradient G_0, which depends of the anisotropic restricted-diffusion weighting. Notice that the second term is always positive and it contains the IGDT $\left\langle\Delta\vec{G}_0\Delta\vec{G}_0\right\rangle$. This was the expression used to evaluate the results presented in Fig. 4 after being normalized by $\Delta\beta^s = \Delta\beta_{G^2}$ to remove the anisotropic weighting due to restricted diffusion effects. For an isotropic diffusion $\mathbf{D}(\omega) = D(\omega)\mathbf{I}$, this gets simplified to

$$\Delta\beta_{\vec{G}\cdot\vec{G}_0} = \Delta\tilde{D}_{\mathrm{iso}}\vec{G}\cdot\left\langle\vec{G}_0\right\rangle + \Delta\tilde{D}^2_{\mathrm{iso}}\vec{G}\cdot\left\langle\Delta\vec{G}_0\Delta\vec{G}_0\right\rangle\cdot\vec{G}. \quad \text{(S.25)}$$

where $\Delta\tilde{D}_{\mathrm{iso}} = \frac{\gamma^2}{2}\int_{-\infty}^{\infty} d\omega\, 2\mathrm{Re}\left\{\left[F^{aNOGSE}(\omega,TE) - F^{sNOGSE}(\omega,TE)\right]^{\dagger} D(\omega)\,F_0\left(\omega,TE\right)\right\}$.

Supporting Information 3: sNOGSE/aNOGSE analytical expressions for the case of free, unrestricted diffusion

For free diffusion only the tail of the displacement power spectrum $D(\omega) \propto 1/\omega^2$ will be important, as it is this short-times regime active before restriction effects are seen, that matters [20, 44]. The purely applied-gradient diffusion term $M_{G^2}^{\binom{s}{a}NOGSE}(TE)$ is then given by [44]

$$M_{G^2}^{\binom{\mathrm{s}}{\mathrm{a}}\mathrm{NOGSE}}(TE) = \exp\left\{-\frac{1}{12}\gamma^2 G^2 D_0 \left[(N-2)\, x^3 + 2y^3\right]\right\}, \tag{S.26}$$

where $(N-2)\, x + 2y = TE_{NOGSE} = TE/2$ (see Fig. 2 of the main text for definitions). The pure background gradient decay term is in turn

$$M_{G_0^2}(TE, N) = \exp\left\{-\frac{1}{12}\gamma^2 G_0^2 D_0 TE^3\right\}, \tag{S.27}$$

which is independent of x and y, and therefore of the applied gradient modulation as was mentioned in the manuscript.

The cross-term signal-decay contribution for sNOGSE is zero as described before, and the one for aNOGSE will be

$$M_{\vec{G}\cdot\vec{G}_0}^{aNOGSE}(TE) = \exp\left\{-\frac{1}{4}\gamma^2 \vec{G}\cdot\vec{G}_0 D_0 TE_{NOGSE}\, (-1)^{N/2}\left[2y^2 - \left(1 + (-1)^{N/2}\right) x^2\right]\right\}. \tag{S.28}$$

Notice that the sign of the attenuation factor for this cross-term contribution depends of the relative sign of $G_{\|} G_0$, where $G_{\|}$ is the applied component of the G-gradient that is parallel to the background gradient vector. The extremes of this attenuation arise for $x = y = TE_{NOGSE}/N$ (CPMG-like modulation)

$$\beta_{\vec{G}\cdot\vec{G}_0}^{CPMG}(TE) = -\frac{1}{4}\gamma^2 \vec{G}\cdot\vec{G}_0 D_0 \frac{TE_{NOGSE}^3}{N^2}\left((-1)^{N/2} - 1\right), \tag{S.29}$$

and for $y = TE_{NOGSE}/2$ and $x = 0$ (single-echo modulation)

$$\beta_{\vec{G}\cdot\vec{G}_0}^{Single-echo}(TE) = -\frac{1}{8}\gamma^2 \vec{G}\cdot\vec{G}_0 D_0 TE_{NOGSE}^3\, (-1)^{N/2}. \tag{S.30}$$

Given the $(-1)^{N/2}$ factor in Eq. (S.28) it follows that if $N/2$ is even, $\beta_{\vec{G}\cdot\vec{G}_0}^{CPMG}(TE) = 0$ and the contrast contribution for the difference of attenuation factors is

$$\Delta\beta_{\vec{G}\cdot\vec{G}_0} = \frac{1}{8}\gamma^2 \vec{G}\cdot\vec{G}_0 D_0 TE_{NOGSE}^3; \tag{S.31}$$

i.e., it depends on the relative sign of $G_{\|}$ and G_0. If $N/2$ is odd $\beta^{CPMG}_{\vec{G}\cdot\vec{G}_0}(TE) \neq 0$; but for $N/2$ odd the attenuation decays with $1/N^2$, and this makes the contrast lower. Assuming a Gaussian distribution for G_0,

$$\beta_{\vec{G}\cdot\vec{G}_0}(TE) = -\frac{1}{4}\gamma^2 D_0 TE_{NOGSE}\,(-1)^{N/2}\,(2y^2 - \left(1 + (-1)^{N/2}\right)x^2)\,\vec{G}\cdot\left\langle\vec{G}_0\right\rangle + \frac{1}{32}\gamma^4 D_0^2 TE^2_{NOGSE}\left[2y^2 - \left(1+(-1)^{N/2}\right)x^2\right]^2\left\langle\left[\vec{G}\cdot\left(\vec{G}_0 - \left\langle\vec{G}_0\right\rangle\right)\right]^2\right\rangle. \quad \text{(S.32)}$$

The attenuation factor contrast amplitude is then

$$\Delta\beta_{\vec{G}\cdot\vec{G}_0} = \frac{1}{8}\gamma^2 D_0 TE^3_{NOGSE}\,\vec{G}\cdot\left\langle\vec{G}_0\right\rangle + \frac{1}{128}\gamma^4 D_0^2 TE^6_{NOGSE}\,\vec{G}\cdot\left\langle\Delta\vec{G}_0\Delta\vec{G}_0\right\rangle\cdot\vec{G}, \quad \text{(S.33)}$$

where $\left\langle\Delta\vec{G}_0\Delta\vec{G}_0\right\rangle$ is the IGDT.

---

[1] Callaghan, P. T. *Principles of Nuclear Magnetic Resonance Microscopy* (Oxford University Press, 1993).

[2] Price, W. S. Pulsed-field gradient nuclear magnetic resonance as a tool for studying translational diffusion: Part 1. basic theory. *Concepts Magn. Reson.* **9**, 299–336 (1997).

[3] Sen, P. N. Time-dependent diffusion coefficient as a probe of geometry. *Concepts Magn. Reson.* **23**, 1–21 (2004).

[4] Grebenkov, D. S. NMR survey of reflected brownian motion. *Rev. Mod. Phys.* **79**, 1077–1137 (2007).

[5] Gore, J. C. *et al.* Characterization of tissue structure at varying length scales using temporal diffusion spectroscopy. *NMR in Biomedicine* **23**, 745–756 (2010).

[6] Mitra, P. P., Sen, P. N., Schwartz, L. M. & Le Doussal, P. Diffusion propagator as a probe of the structure of porous media. *Phys. Rev. Lett.* **68**, 3555–3558 (1992).

[7] Basser, P., Mattiello, J. & LeBihan, D. *J. Magn. Reson., Series B* **103**, 247 (1994).

[8] Basser, P., Mattiello, J. & LeBihan, D. MR diffusion tensor spectroscopy and imaging. *Biophys. J.* **66**, 259–267 (1994).

[9] Kukla, V. *et al.* NMR studies of single-file diffusion in unidimensional channel zeolites. *Science* **272**, 702–704 (1996).

[10] Kuchel, P. W., Coy, A. & Stilbs, P. NMR "diffusion-diffraction" of water revealing alignment of erythrocytes in a magnetic field and their dimensions and membrane transport characteristics. *Magn. Reson. Med.* **37**, 637–643 (1997).

[11] Mair, R. W. *et al.* Probing porous media with gas diffusion NMR. *Phys. Rev. Lett.* **83**, 3324–3327 (1999).

[12] Peled, S., Cory, D. G., Raymond, S. A., Kirschner, D. A. & Jolesz, F. A. Water diffusion, t2, and compartmentation in frog sciatic nerve. *Magn. Reson. Med.* **42**, 911–918 (1999).

[13] Song, Y.-Q., Ryu, S. & Sen, P. N. Determining multiple length scales in rocks. *Nature* **406**, 178–181 (2000).

[14] LeBihan, D. Looking into the functional architecture of the brain with diffusion MRI. *Nat. Rev. Neurosci.* **4**, 469–480 (2003).

[15] Song, Y.-Q., Zielinski, L. & Ryu, S. Two-dimensional NMR of diffusion systems. *Phys. Rev. Lett.* **100**, 248002 (2008).

[16] Lawrenz, M. & Finsterbusch, J. Double-wave-vector diffusion-weighted imaging reveals microscopic diffusion anisotropy in the living human brain. *Magn. Reson. Med.* **69**, 1072–1082 (2013).

[17] Hertel, S. A. *et al.* Magnetic-resonance pore imaging of nonsymmetric microscopic pore shapes. *Phys. Rev. E* **92**, 012808 (2015).

[18] Ong, H. H. *et al.* Indirect measurement of regional axon diameter in excised mouse spinal cord with q-space imaging: simulation and experimental studies. *Neuroimage* **40**, 1619–1632 (2008).

[19] Budde, M. D. & Frank, J. A. Neurite beading is sufficient to decrease the apparent diffusion coefficient after ischemic stroke. *Proc. Natl. Acad. Sci. U. S. A.* **107**, 14472–14477 (2010).

[20] Álvarez, G. A., Shemesh, N. & Frydman, L. Coherent dynamical recoupling of diffusion-driven decoherence in magnetic resonance. *Phys. Rev. Lett.* **111**, 080404 (2013).

[21] Shemesh, N., Álvarez, G. A. & Frydman, L. Measuring small compartment dimensions by probing diffusion dynamics via non-uniform oscillating-gradient spin-echo (NOGSE) NMR. *J. Magn. Reson.* **237**, 49–62 (2013).

[22] Assaf, Y. & Basser, P. J. Composite hindered and restricted model of diffusion (CHARMED) MR imaging of the human brain. *Neuroimage* **27**, 48–58 (2005).

[23] Panagiotaki, E. *et al.* Compartment models of the diffusion MR signal in brain white matter:

A taxonomy and comparison. *Neuroimage* **59**, 2241–2254 (2012).

[24] Warren, W. S. *et al.* MR Imaging Contrast Enhancement Based on Intermolecular Zero Quantum Coherences. *Science* **281**, 247 –251 (1998).

[25] Sen, P. & Axelrod, S. Inhomogeneity in local magnetic field due to susceptibility contrast. *J. Appl. Phys.* **86**, 4548–4554 (1999).

[26] Faber, C., Pracht, E. & Haase, A. Resolution enhancement in in vivo NMR spectroscopy: detection of intermolecular zero-quantum coherences. *J. Magn. Reson.* **161**, 265–274 (2003).

[27] Pathak, A., Ward, B. & Schmainda, K. A novel technique for modeling susceptibility-based contrast mechanisms for arbitrary microvascular geometries: The finite perturber method. *Neuroimage* **40**, 1130–1143 (2008).

[28] Wharton, S. & Bowtell, R. Whole-brain susceptibility mapping at high field: A comparison of multiple- and single-orientation methods. *Neuroimage* **53**, 515–525 (2010).

[29] Lee, J. *et al.* Sensitivity of MRI resonance frequency to the orientation of brain tissue microstructure. *Proc. Natl. Acad. Sci. U. S. A.* **107**, 5130–5135 (2010).

[30] de Rochefort, L. *et al.* Quantitative susceptibility map reconstruction from MR phase data using bayesian regularization: Validation and application to brain imaging. *Magn. Reson. Med.* **63**, 194–206 (2010).

[31] Li, W., Wu, B., Avram, A. & Liu, C. Magnetic susceptibility anisotropy of human brain in vivo and its molecular underpinnings. *Neuroimage* **59**, 2088–2097 (2012).

[32] Liu, C. & Li, W. Imaging neural architecture of the brain based on its multipole magnetic response. *Neuroimage* **67**, 193–202 (2013).

[33] Chen, W., Foxley, S. & Miller, K. Detecting microstructural properties of white matter based on compartmentalization of magnetic susceptibility. *Neuroimage* **70**, 1–9 (2013).

[34] Liu, C. Susceptibility tensor imaging. *Magn. Reson. Med.* **63**, 1471–1477 (2010).

[35] Lee, J. *et al.* T-2*-based fiber orientation mapping. *Neuroimage* **57**, 225–234 (2011).

[36] Oh, S., Kim, Y., Cho, Z. & Lee, J. Origin of b-0 orientation dependent r*(2) (=1/T*(2)) in white matter. *Neuroimage* **73**, 71–79 (2013).

[37] Liu, C., Li, W., Wu, B., Jiang, Y. & Johnson, G. 3D fiber tractography with susceptibility tensor imaging. *Neuroimage* **59**, 1290–1298 (2012).

[38] Haacke, E., Xu, Y., Cheng, Y. & Reichenbach, J. Susceptibility weighted imaging (SWI). *Magn. Reson. Med.* **52**, 612–618 (2004).

[39] Thomas, B. P. *et al.* High-Resolution 7t MRI of the Human Hippocampus In Vivo. *J. Magn. Reson. Imaging* **28**, 1266–1272 (2008).

[40] Yao, B. *et al.* Susceptibility contrast in high field MRI of human brain as a function of tissue iron content. *Neuroimage* **44**, 1259–1266 (2009).

[41] Shmueli, K. *et al.* Magnetic susceptibility mapping of brain tissue in vivo using MRI phase data. *Magn. Reson. Med.* **62**, 1510–1522 (2009).

[42] Haacke, E. M., Mittal, S., Wu, Z., Neelavalli, J. & Cheng, Y.-C. N. Susceptibility-Weighted Imaging: Technical Aspects and Clinical Applications, Part 1. *Am. J. Neuroradiology* **30**, 19–30 (2009).

[43] Han, S. *et al.* Magnetic field anisotropy based MR tractography. *J. Magn. Reson.* **212**, 386–393 (2011).

[44] Álvarez, G. A., Shemesh, N. & Frydman, L. Diffusion-assisted selective dynamical recoupling: A new approach to measure background gradients in magnetic resonance. *J. Chem. Phys.* **140**, 084205 (2014).

[45] Stepisnik, J. Time-dependent self-diffusion by NMR spin-echo. *Physica B* **183**, 343–350 (1993).

[46] Callaghan, P. T. & Stepisnik, J. Frequency-domain analysis of spin motion using modulated-gradient NMR. *J. Magn. Reson.* **117**, 118–122 (1995).

[47] Stepisnik, J., Lasic, S., Mohoric, A., Sersa, I. & Sepe, A. Spectral characterization of diffusion in porous media by the modulated gradient spin echo with CPMG sequence. *J. Magn. Reson.* **182**, 195–199 (2006).

[48] Stepisnik, J. Validity limits of gaussian approximation in cumulant expansion for diffusion attenuation of spin echo. *Physica B* **270**, 110–117 (1999).

[49] Zheng, G. & Price, W. Suppression of background gradients in (b-0 gradient-based) NMR diffusion experiments. *Concepts Magn. Reson.* **30A**, 261–277 (2007).

[50] Cho, H., Ryu, S., Ackerman, J. & Song, Y. Visualization of inhomogeneous local magnetic field gradient due to susceptibility contrast. *J. Magn. Reson.* **198**, 88–93 (2009).
